# BUILDING CAPACITY TO ADDRESS HOARDING AND CLUTTER AMONG OLDER ADULTS: DEVELOPING A NETWORK OF TRAINED PROVIDERS

**DOI:** 10.1093/geroni/igae098.3100

**Published:** 2024-12-31

**Authors:** Bronwyn Keefe, Megan Nizza, Jordana Muroff

**Affiliations:** Boston University, Boston, Massachusetts, United States; Boston University, Boston, Massachusetts, United States; Boston University, Boston, Massachusetts, United States

## Abstract

Hoarding disorder (HD) is a challenging mental health and public health problem characterized by difficulty discarding possessions, and excessive acquisition that contribute to substantial clutter and impaired functioning. HD occurs in 2-5% of adults and worsens with age. Clutter limits the ability to utilize space and function in a home, poses significant health risks, and creates housing instability. The lack of people trained in HD limits access to evidence-based assessments and interventions and home-based support that may mitigate risks. To address this, the Center for Aging and Disability Education and Research developed an online course on Hoarding Disorder in Older Adults to increase the knowledge and skills of those working in community-based agencies, including housing and local health departments. A total of 110 people completed the training, with 96% strongly agreeing it expanded their knowledge and understanding and 93% saying this training will help them to apply new practice skills. The learners showed significant improvements (p<.001) in all learning competencies from the pre-post-course self-assessments. The largest change was in the area of being able to identify intervention options and current treatment approaches with hoarding. This program can serve as a model to improve the attitudes, knowledge, and practice behaviors of a wide range of people who work with older adults. The goal is that this will lead to successfully engaging those with hoarding, which may increase openness to services, improve their mental health, and reduce public health risks so older adults can continue to live independently.

